# The longevity gene mIndy (I’m Not Dead, Yet) affects blood pressure through sympathoadrenal mechanisms

**DOI:** 10.1172/jci.insight.136083

**Published:** 2021-01-25

**Authors:** Diana M. Willmes, Martin Daniels, Anica Kurzbach, Stefanie Lieske, Nicole Bechmann, Tina Schumann, Christine Henke, Nermeen N. El-Agroudy, Andrey C. Da Costa Goncalves, Mirko Peitzsch, Anja Hofmann, Waldemar Kanczkowski, Kristin Kräker, Dominik N. Müller, Henning Morawietz, Andreas Deussen, Michael Wagner, Ali El-Armouche, Stephen L. Helfand, Stephan R. Bornstein, Rafael de Cabo, Michel Bernier, Graeme Eisenhofer, Jens Tank, Jens Jordan, Andreas L. Birkenfeld

**Affiliations:** 1 Section of Metabolic and Vascular Medicine, Medical Clinic III, University Hospital and Medical Faculty Carl Gustav Carus and; 2 Paul Langerhans Institute Dresden of the Helmholtz Center Munich at University Hospital and Faculty of Medicine, Technical University Dresden, Dresden, Germany.; 3 German Center for Diabetes Research (DZD e.V.), Neuherberg, Germany.; 4 Institute of Diabetes Research and Metabolic Diseases (IDM) of the Helmholtz Center Munich, University of Tübingen, Tübingen, Germany.; 5 Department of Internal Medicine IV, Endocrinology, Diabetology and Nephrology, University Hospital Tübingen, Tübingen, Germany.; 6 Department of Diabetes, School of Life Course Science, Faculty of Life Sciences & Medicine, King’s College London, London, United Kingdom.; 7 Institute of Clinical Chemistry and Laboratory Medicine, University Hospital and Medical Faculty Carl Gustav Carus, Technical University Dresden, Dresden, Germany.; 8 Leibniz Institute for Molecular Pharmacology (FMP), Campus Berlin-Buch, Berlin, Germany.; 9 Division of Vascular Endothelium and Microcirculation, Medical Clinic III, University Hospital and Medical Faculty Carl Gustav Carus, Technical University Dresden, Dresden, Germany.; 10 Experimental and Clinical Research Center, Max Delbruck Center for Molecular Medicine and Charité – University Hospital Berlin, Berlin, Germany.; 11 Department of Physiology, Medical Faculty Carl Gustav Carus, and; 12 Department of Pharmacology and Toxicology, University Hospital and Medical Faculty Carl Gustav Carus, Technical University Dresden, Dresden, Germany.; 13 Department of Molecular Biology, Cell Biology & Biochemistry, Division of Biology and Medicine, Brown University, Providence, Rhode Island, USA.; 14 Translational Gerontology Branch, National Institute on Aging (NIA), National Institutes of Health (NIH), Baltimore, Maryland, USA.; 15 Aerospace Medicine, University of Cologne, Cologne, Germany.; 16 Institute for Aerospace Medicine, German Aerospace Center (DLR), Cologne, Germany.

**Keywords:** Metabolism, Vascular Biology, Cardiovascular disease

## Abstract

Reduced expression of the plasma membrane citrate transporter INDY (acronym *I’m Not Dead, Yet*) extends life span in lower organisms. Deletion of the mammalian *Indy* (*mIndy*) gene in rodents improves metabolism via mechanisms akin to caloric restriction, known to lower blood pressure (BP) by sympathoadrenal inhibition. We hypothesized that *mIndy* deletion attenuates sympathoadrenal support of BP. Continuous arterial BP and heart rate (HR) were reduced in mINDY-KO mice. Concomitantly, urinary catecholamine content was lower, and the decreases in BP and HR by mIndy deletion were attenuated after autonomic ganglionic blockade. Catecholamine biosynthesis pathways were reduced in mINDY-KO adrenals using unbiased microarray analysis. Citrate, the main mINDY substrate, increased catecholamine content in pheochromocytoma cells, while pharmacological inhibition of citrate uptake blunted the effect. Our data suggest that deletion of *mIndy* reduces sympathoadrenal support of BP and HR by attenuating catecholamine biosynthesis. Deletion of *mIndy* recapitulates beneficial cardiovascular and metabolic responses to caloric restriction, making it an attractive therapeutic target.

## Abstract 2

Deletion of mIndy reduces blood pressure and heart rate by attenuating catecholamine biosynthesis and recapitulates beneficial cardiovascular and metabolic responses to caloric restriction.

## Graphical abstract

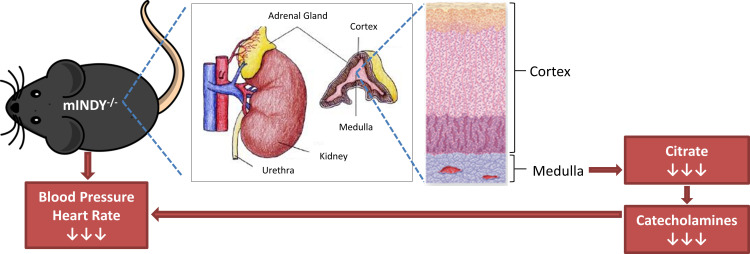

## Introduction

A close correlation exists between energy balance, BP regulation, and life expectancy. Caloric excess promotes obesity, arterial hypertension, and premature mortality, whereas reducing dietary calories decreases adiposity and arterial BP while extending mean and maximum life span in diverse species (1–3). Moreover, caloric restriction protects from and reverses nutritionally induced and aging-related adiposity and arterial hypertension in rodents, primates, and human subjects (1, 2). Physiological mechanisms mediating the beneficial effects of reduced calorie availability on BP regulation include reduced activity of the sympathetic nervous system and renin-angiotensin-aldosterone system, in concert with increased mitochondrial biogenesis (1, 2, 4). These data suggest specific links between metabolic and BP regulation. One of these links may be the mammalian *Indy* gene (*mIndy,* acronym *I’m Not Dead, Yet*), which codes for a plasma membrane citrate transporter, regulating energy homeostasis and mitochondrial function (5).

Reduced *Indy* gene expression prolongs life in *Drosophila melanogaster* and *Caenorhabditis elegans* by mechanisms mimicking caloric restriction (6, 7). We generated a mammalian *Indy*-knockout model, the mINDY-KO mouse, addressing the effect of *mIndy* on mammalian physiology. Deletion of *mIndy* protected mice from many characteristics of the cardiometabolic syndrome that develop with high-calorie feeding and aging, including adiposity, nonalcoholic fatty liver, insulin resistance, and mitochondrial dysfunction (8). Similarly, knockdown of *mIndy* in rats and mice improved glucose and lipid homeostasis (9, 10). Moreover, unbiased, whole-genome, microarray-based analysis of hepatic transcriptional regulation of ad libitum–fed mINDY-KO mice showed that 80% of pathways were similarly regulated compared with caloric restricted wild-type (WT) control mice (8). These data indicate important functional and transcriptional similarities between *mIndy* deletion and caloric restriction. We tested the hypothesis that similar to caloric restriction, loss of *mIndy* reduces arterial BP and heart rate (HR) by decreasing the sympathoadrenal support in mice independently of body composition. Radiotelemetry was used to evaluate the effect of *mIndy* deletion on BP and HR in young, age-matched, male mINDY-KO and WT mice fed a normal chow diet, without differences in body composition. Moreover, we assessed autonomic cardiovascular control using state-of-the-art physiological, biochemical, and pharmacological profiling and studied the effect of the deletion of mINDY in adrenal glands, ex vivo and in cellular culture in vitro.

## Results

### Deletion of mIndy leads to reduced body weight and size but normal body composition.

**Figure 1:**
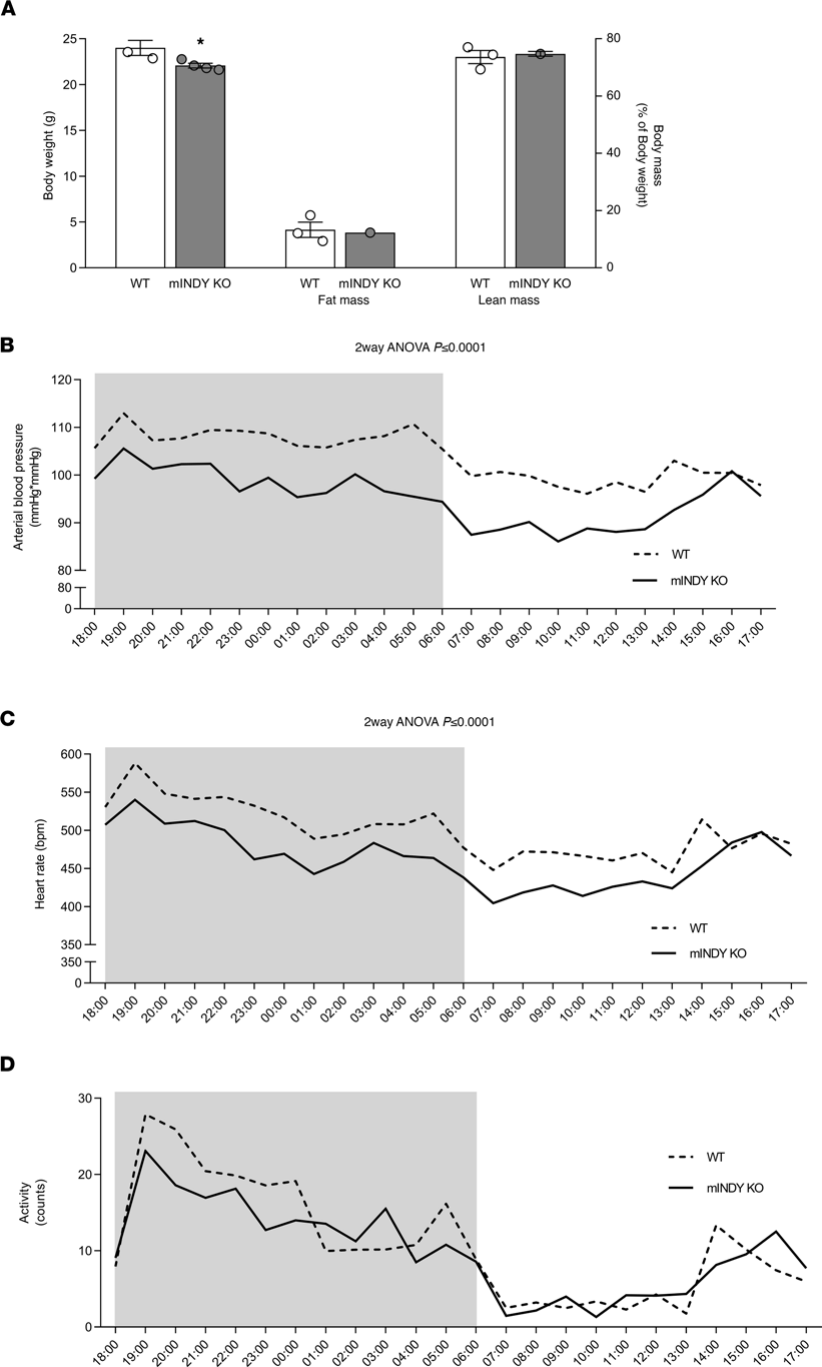
BW and body composition, arterial BP, HR, and voluntary activity of mINDY-KO mice and WT littermate controls. (**A**) After a 16-hour fast, BW and body composition were measured in 13- to 14-week-old mINDY-KO (*n* = 4) and WT littermate control (*n* = 3) mice fed a regular chow diet. BW and body composition were as follows: mINDY-KO (25.2 ± 0.3 g; 74.74% lean mass and 12.28% fat mass of BW); WT littermates (27.6 ± 1.2 g; 73.6% lean mass and 13.3% fat mass of BW). mINDY-KO mice had a lower BW based on a shorter body length compared with WT littermate controls. A 2-tailed Student’s *t* test was performed to determine significance level between groups. Data represent the mean ± SEM (*P < 0.05). (**B**–**D**) Radiotelemetry system was used for monitoring arterial BP, HR, and voluntary activity in mINDY-KO (*n* = 6) and WT littermate control mice (*n* = 8) fed a regular chow diet. (**B**) Arterial BP (mINDY-KO overall 96 ± 2 mmHg; day, 92 ± 3 mmHg, and night, 99 ± 3 mmHg. WT overall 104 ± 2 mmHg; day, 100 ± 3 mmHg, and night, 108 ± 1 mmHg). (**C**) HR (mINDY-KO overall 463 ± 7 bpm; day, 443 ± 9 bpm, and night, 484 ± 10 bpm. WT overall 500 ± 5 bpm; day, 474 ± 5 bpm, and night, 526 ± 5 bpm). (**D**) Voluntary activity (mINDY-KO overall 10 ± 1 counts/min; day, 5 ± 1 counts/min, and night, 15 ± 2 counts/min. WT overall 11 ± 1 counts/min; day, 5 ± 1 counts/min, and night, 17 ± 1 counts/min). Mean daily values from all animals during a 3-day period were analyzed. A 2-way ANOVA was performed to determine significance level.

mINDY-KO mice were generated as previously described (8). Heterozygous mINDY-KO mice were bred and litters contained the expected ratio of WT and homozygous mINDY-KO mice. Compared with WT littermate controls, mINDY-KO mice on a regular chow diet had an approximately 10% lower body weight (BW) at the age of 13–14 weeks after a 16-hour fast (Figure 1A), due to a reduction in body size (Supplemental Figure 1A; supplemental material available online with this article; https://doi.org/10.1172/jci.insight.136083DS1). Fat mass and lean mass did not differ between genotypes, as shown by NMR spectroscopy (Figure 1A). These results confirm our previous data (8).

### mIndy deletion reduces arterial BP and HR.

In order to determine BP, radiotelemetric probes were implanted into the right femoral artery of 12-week-old mice of both genotypes. Implantation in the femoral artery spares carotid and aortic baroreceptors, which are crucial for autonomic cardiovascular control. Animals were allowed to recover for 7 days postimplantation, and then intra-arterial BP was assessed over a 3-day period. Mean arterial BP was reduced in mINDY-KO mice by 8 mmHg compared with littermate WT mice (Figure 1B and Table 1), with similar reductions in diastolic and systolic BP (Supplemental Figure 1, B and C, and Table 1). At the same time, HR was reduced by 37 bpm in mINDY-KO mice (Figure 1C and Table 1). To rule out that the intra-arterial probe in the femoral artery introduced a bias in physical activity, we assessed locomotion and observed that activity was similar between genotypes (Figure 1D and Table 1).

### mIndy deletion reduces autonomic support of BP and baroreflex sensitivity.

**Figure 2:**
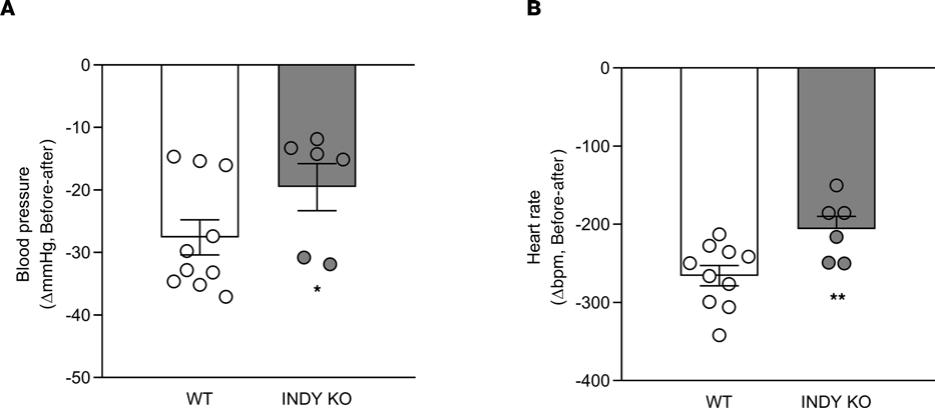
BP and HR in response to trimethaphan. mINDY-KO (*n* = 3) and littermate WT control mice (*n* = 5) were treated with trimethaphan (120 mg/kg BW) followed by the measurement of (**A**) individual BP response (mINDY-KO, –34 ± 10 ΔmmHg; WT, 28 ± 3 ΔmmHg) and (**B**) HR (mINDY-KO, –210 ± 15 Δbpm; WT, –266 ± 13 Δbpm). Changes in HR and BP are expressed as change relative to baseline (measured over a time frame of 1200 seconds). Values of 2 different time frames of 1200 seconds each (2400 seconds in total) were analyzed per animal. A 2-tailed Student’s *t* test was performed to determine significance level between groups. Data represent the mean ± SEM (*P < 0.05; **P < 0.01).

Concomitant reductions in arterial BP and HR suggest autonomic nervous system involvement, specifically a decrease in sympathoadrenal tone. Ganglionic blockade with trimethaphan decreased BP (WT = –28 ± 3 ΔmmHg; mINDY-KO = –20 ± 4 ΔmmHg; *P* < 0.05; Figure 2A) and HR (WT = –266 ± 13 Δbpm; mINDY-KO = –206 ± 16 Δbpm; *P* < 0.01; Figure 2B) to a lower extent in mINDY-KO versus littermate WT controls. These in vivo findings are consistent with reduced autonomic support of BP through the sympathetic nervous system with reduction in adrenergic vascular tone and perhaps cardiac innervation in mINDY-KO mice.

**Figure 3:**
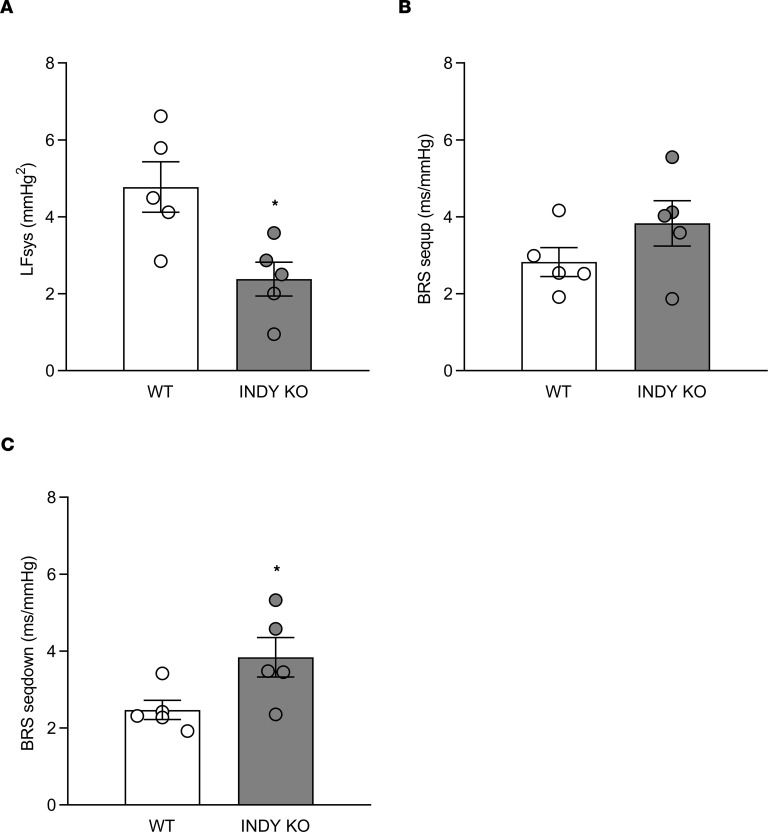
Systolic BP variability and baroreflex sensitivity. Systolic BP variability in the frequency range and baroreflex sensitivity (BRS) were calculated by cross-spectral analysis (BRS-LF) or by the sequence technique (BRS sequp, BRS seqdown) in mINDY-KO (*n* = 5) and littermate WT controls (*n* = 5) at the age of 3–4 months. Time length measurement was similar between genotypes (WT, 3665 ± 137 s; mINDY-KO, 3694 ± 133 s). (**A**) LFsys (WT, 4.8 ± 0.7 mmHg^2^; mINDY-KO, 2.4 ± 0.4 mmHg^2^); (**B**) BRS sequp (WT, 2.8 ± 0.4 ms/mmHg; mINDY-KO, 3.8 ± 0.6 ms/mmHg). (**C**) BRS seqdown (WT, 2.5 ± 0.3 ms/mmHg; mINDY-KO, 3.8 ± 0.5 ms/mmHg). Significance level was determined by a 2-tailed Student’s *t* test. Data represent the mean ± SEM (*P < 0.05).

Dynamic influences of sympathetic and parasympathetic activity on cardiovascular control can be elucidated through BP variability and baroreflex sensitivity measurements. Systolic BP variability in the low-frequency band was reduced by 50% with mINDY deletion (LFsys; WT = 4.8 ± 0.6 mmHg^2^; mINDY-KO = 2.4 ± 0.4 mmHg^2^; Figure 3A; *P* < 0.05). The increase in LFsys in obese mouse models is characterized by increased sympathetic drive (11). Moreover, rapid baroreflex-mediated HR changes in mice are primarily mediated through cardiac vagal efferents. Measures that augment vagal activity are associated with improved baroreflex sensitivity (11, 12). Here, BRS measured by cross-spectral analysis and determined by the sequence method was greater in mINDY-KO compared with WT mice (BRS sequp; WT = 2.8 ± 0.4 ms/mmHg; mINDY-KO = 3.8 ± 0.6 ms/mmHg; Figure 3B; BRS seqdown; WT = 2.5 ± 0.3 ms/mmHg; mINDY-KO = 3.8 ± 0.5 ms/mmHg; Figure 3C; *P* < 0.05). These findings strongly support the idea that mINDY deletion is associated with a shift of cardiovascular sympathetic and parasympathetic modulation toward parasympathetic predominance.

### mIndy deletion attenuates urinary catecholamines and catechloaminergic pathways of the adrenal gland transcriptome.

**Figure 4:**
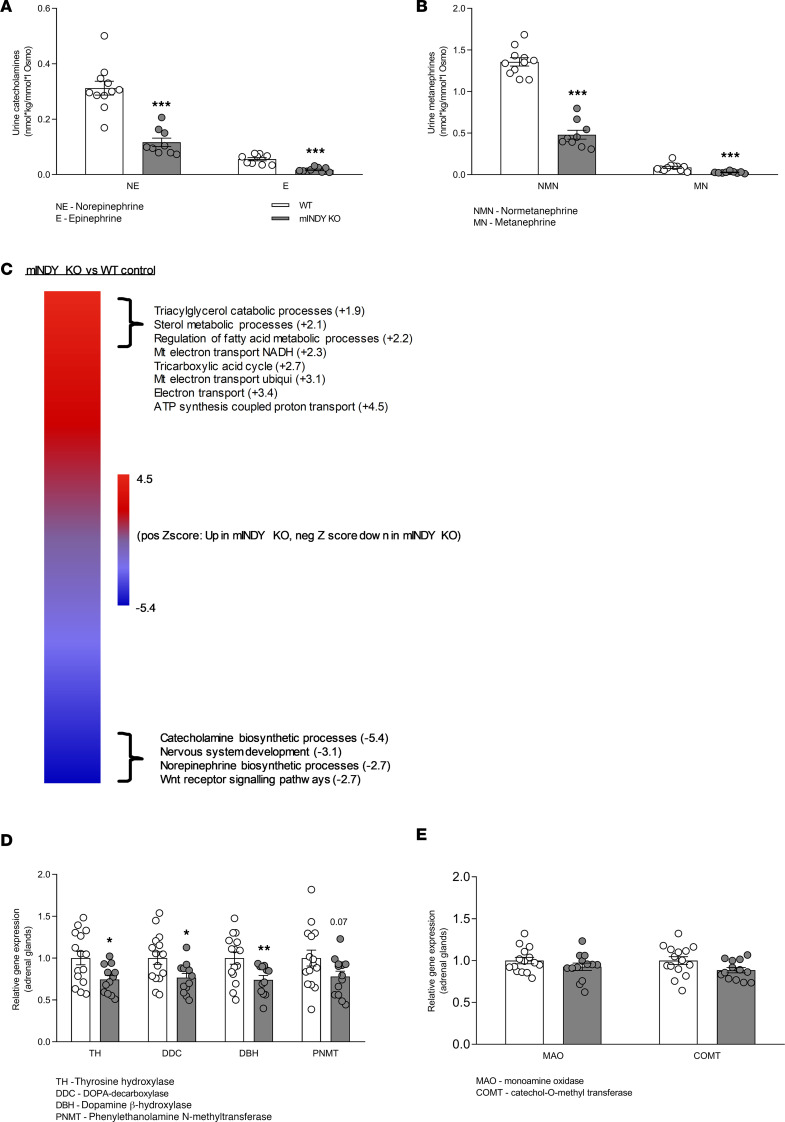
Urinary catecholamines, metanephrines, and adrenal gland transcriptomics. (**A**) Urinary secretion of NE and E in mINDY-KO (*n* = 9; NE, 0.12 ± 0.02, and E, 0.02 ± 0.003 nmol × kg/mmol × L Osmo) and WT littermate control mice (*n* = 9–11; NE, 0.31 ± 0.02, and E, 0.06 ± 0.006 nmol × kg/mmol × L Osmo). (**B**) Urinary secretion of NMN and MN in mINDY-KO (*n* = 9; NMN, 0.48 ± 0.05, and MN, 0.03 ± 0.004 nmol × kg/mmol × L Osmo) and WT littermate control mice (*n* = 11; NMN, 1.36 ± 0.05, and MN, 0.09 ± 0.014 nmol × kg/mmol × L Osmo). (**C**) Adrenal gland gene set enrichment analysis in mINDY-KO (*n* = 5) and littermate control mice (*n* = 6) fed a regular chow diet. (**D**) Relative gene expression of catecholamine biosynthetic enzymes and (**E**) enzymes involved in catecholamine degradation in adrenal glands of mINDY-KO (*n* = 12–13) and WT littermate controls (*n* = 15). Significance level was determined by a 2-tailed Student’s *t* test. Data represent the mean ± SEM (*P < 0.05; **P < 0.01; ***P < 0.001).

We further investigated the sympathoadrenal system as a mediator of the beneficial cardiovascular effect of mINDY deletion. The sympathetic nervous system regulates arterial BP and HR via norepinephrine released from postganglionic adrenergic terminals (13, 14). Norepinephrine (NE) and epinephrine (E) and their respective O-methylated metabolites, normetanephrine (NMN) and metanephrine (MN), can be measured in the circulation or in urine samples collected during a 24-hour period. Because of difficulties in obtaining stress-free peripheral blood, 24-hour urinary catecholamine levels were measured and normalized to urine osmolality. Levels of NE were reduced by 39% and E by 33% in mINDY-KO mice compared with WT littermates (Figure 4A). Urinary NMN and MN outputs were also significantly reduced in mINDY-KO mice (Figure 4B), strongly indicating lower sympathetic support of BP with *mIndy* deletion. These results prompted us to perform whole-genome microarray analysis on adrenal tissue samples of mINDY-KO mice and WT littermates because catecholamines are synthesized and released from the adrenal medulla. Unbiased gene set enrichment analysis revealed markedly decreased expression of pathways regulating catecholamine biosynthesis in mINDY-KO versus WT littermates (Figure 4C). Quantitative real-time PCR (RT-PCR) was used to confirm gene expression results. Important enzymes in catecholamine biosynthesis such as tyrosine hydroxylase (TH) — the rate-limiting step in catecholamine synthesis — DOPA decarboxylase (DDC), dopamine β-hydroxylase (DBH), and phenylethanolamine *N*-methyltransferase (PNMT) showed significant reductions in mINDY-KO mice (Figure 4D). TH reduction was also verified on a protein level (Supplemental Figure 2). In contrast, genes involved in catecholamine degradation like monoamine oxidase (MAO) and catechol-*O*-methyltransferase (COMT) were unaltered between genotypes (Figure 4E).

### Catecholamine biosynthesis is reduced by mIndy deletion.

**Figure 5:**
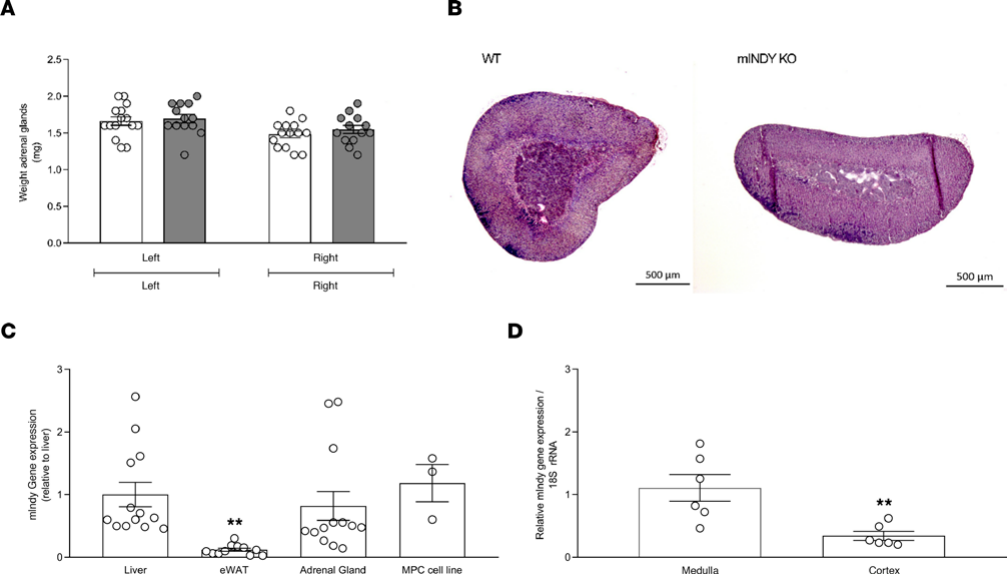
Adrenal gland weight, histology, and *Indy* gene expression. (**A**) Left and right adrenal gland weight in mINDY-KO (*n* =14–15) and WT littermate control (*n* = 13) mice. WT (left, 1.66 ± 0.057 mg; right, 1.48 ± 0.048 mg); mINDY-KO (left, 1.69 ± 0.059 mg; right 1.55 ± 0.055 mg). (**B**) Histological analysis of mINDY-KO (*n* = 3) and WT littermate control mice (*n* = 3). A representative H&E-stained section is shown per genotype. (**C**) Liver, epigonadal white adipose tissue, and adrenal gland *Indy* gene expression in WT mice (*n* = 13). (**D**) Adrenal medulla and cortex *Indy* gene expression in WT mice (*n* = 6). Significance level was determined by a 2-tailed Student’s *t* test in **A** and **D** (**P < 0.01). Significance level was determined by a 1-way ANOVA in **C** (**P < 0.01). Data represent the mean ± SEM.

Catecholamines are synthesized in sympathetic nerve terminals and adrenal medulla (15–19). As a model tissue, adrenal glands were harvested from mINDY-KO mice and littermate controls. Adrenal gland weight (Figure 5A) and histological morphology by H&E staining analysis did not reveal any major differences (Figure 5B). Previous data have suggested that *mIndy* expression is relatively high in the liver as compared with other organs such as skeletal muscle, adipose tissue, and kidneys (20). Interestingly, we found that the level of *mIndy* expression was also high in adrenal tissue (Figure 5C). Here, medullary *Indy* gene expression was about 70% higher than in adrenal cortex (Figure 5D). To confirm effective separation of adrenal medulla from cortex, we determined mRNA expression levels of prototypical medullary and cortical genes. In these samples, relative TH expression was 1.19 ± 0.29 AU in adrenal medulla and 0.11 ± 0.03 AU in adrenal cortex (*P* < 0.01), and steroidogenic acute regulatory protein (StAR) was 0.84 ± 0.07 AU in the adrenal medulla and 3.90 ± 1.23 AU in adrenal cortex (*P* < 0.06) (Supplemental Figure 3). These findings support the idea that reduced catecholamine biosynthesis may have attenuated peripheral catecholamine availability with *mIndy* deletion.

### Inhibition of mINDY transport function in chromaffin pheochromocytoma cells reduces catecholamine content.

**Figure 6:**
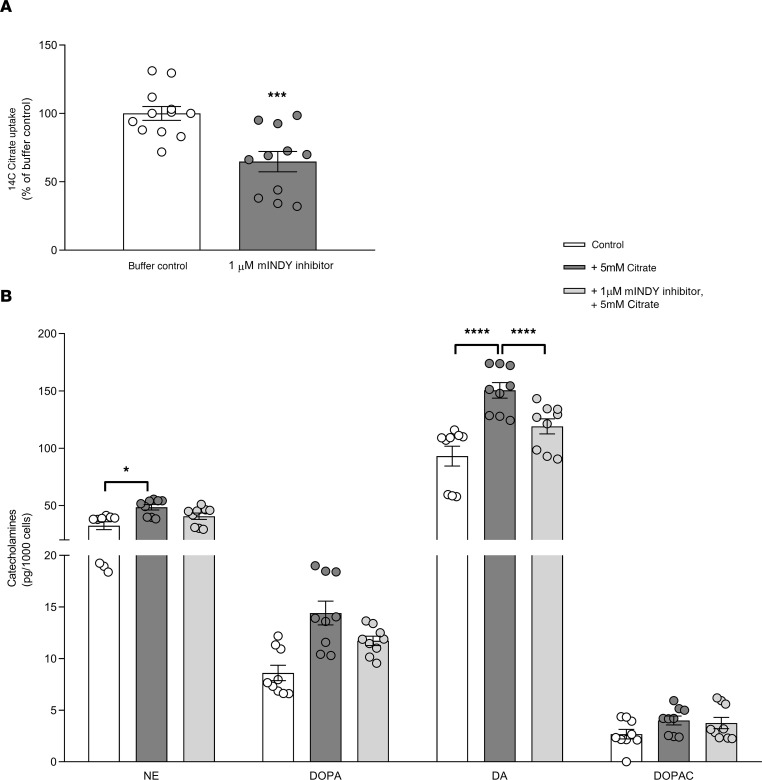
Citrate stimulation and mINDY transport inhibition affect catecholamine biosynthesis in adrenal MPC cells. (**A**) ^14^C-Citrate uptake in the pheochromocytoma cell line MPC treated for 30 minutes with and without 1 μM of the mINDY inhibitor, PF-06761281. (**B**) MPCs were treated with 5 mM sodium citrate or 1 μM PF-06761281, alone or combined, for 72 hours. Lysates were prepared for the determination of NE, 3,4-dihydroxyphenylalanine (DOPA), dopamine (DA), and 3,4-dihydroxyphenylacetic acid (DOPAC) levels. Significance level was determined by a 2-tailed Student’s *t* test in **A** (***P < 0.001). A 2-way ANOVA was performed in **B** to determine significance level between groups (*P < 0.05; ****P < 0.001). Data represent the mean ± SEM.

To test whether inhibition of mINDY transporter function is sufficient to reduce catecholamine content, we treated the chromaffin pheochromocytoma cell line mouse pheochromocytoma cells (MPCs) with the mINDY inhibitor PF-06761281 (21). This small molecule inhibitor reduces mINDY-mediated citrate uptake in mouse hepatocytes with an IC_50_ of 0.21 μM and lowers fasting glucose levels and urinary citrate excretion in high-fat diet–induced obese mice (21). We first assessed citrate uptake in MPCs with and without 1 μM of PF-06761281 and found a significant approximately 35% reduction in the presence of the mINDY inhibitor (*P* < 0.001, Figure 6A). Preincubation of MPC cells with 5 mM sodium citrate increased cellular NE, DOPA, and dopamine content in MPC cells after normalization to cell number, an effect that was partially blunted by addition of 1 μM PF-06761281 before and during citrate treatment (Figure 6B). Previous report showed that PF-06761281 dose-dependently lowers citrate uptake in the liver, kidney, and testis with IC_50_ values of 1.4, 1.0, and 1.0 μM, respectively, while IC_50_ values in human hepatocytes are 0.74 μM and in mouse hepatocytes 0.21 μM (21, 22). Taken together, the reduction in catecholamine content appears to be directly linked to mINDY-mediated citrate transport function. Of note, catecholamine content in absence or presence of the inhibitor was normalized to the viable cell number (Figure 6B) to exclude possible effect of cell viability.

### Steroid hormones, vascular relaxation, and cardiac function are not changed with deletion of mIndy.

**Figure 7:**
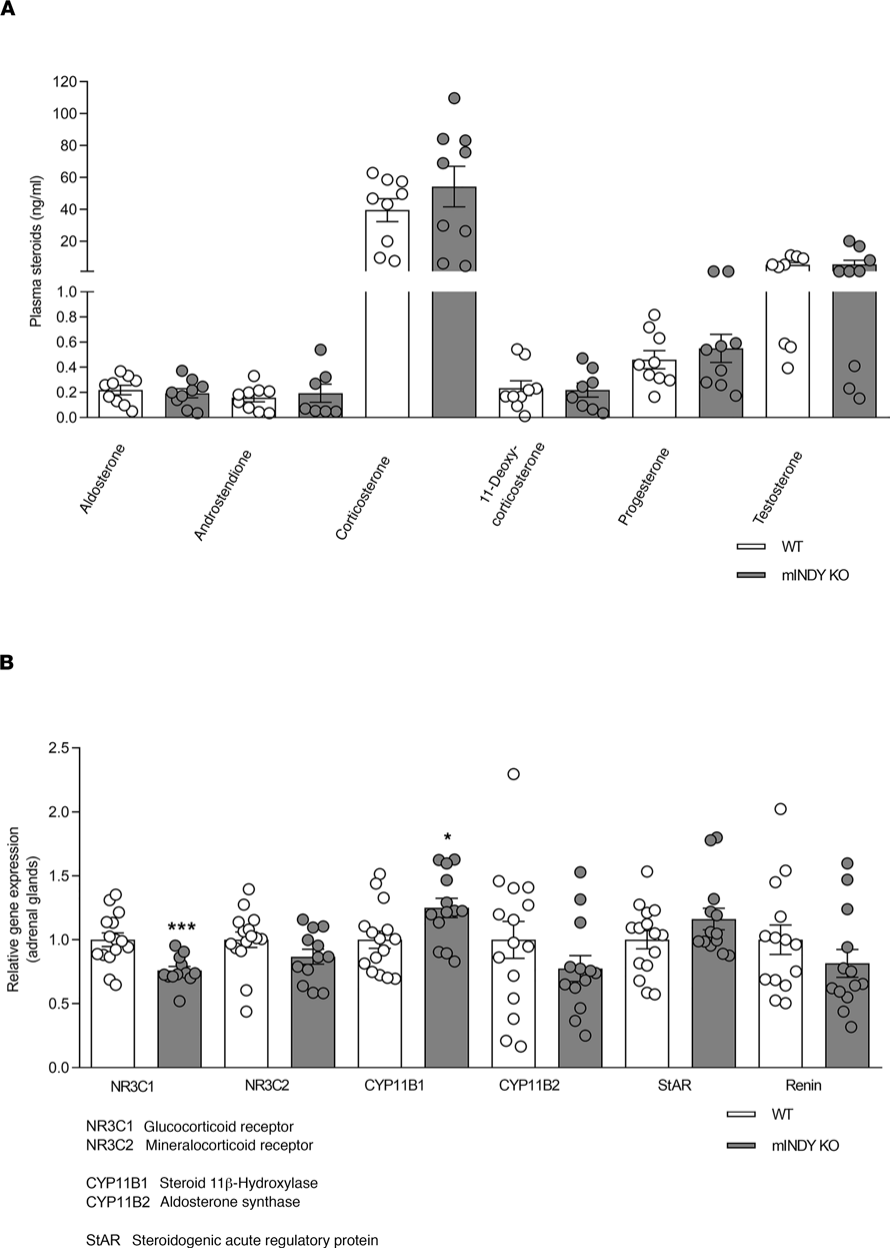
Plasma steroids and steroid hormone synthesis. (**A**) Measurement of circulating steroid hormone concentrations in mINDY-KO (*n* = 9) and WT littermate control mice (*n* = 9). (**B**) Adrenal gland gene expression analysis by RT-PCR of enzymes important for steroid hormone synthesis in mINDY-KO (*n* = 14–15) and WT littermate controls (*n* = 12–13). A 2-tailed Student’s *t* test was performed to determine significance level between groups. Data represent the mean ± SEM (*P < 0.05; ***P < 0.001).

Adrenal glands also synthesize steroid hormones, such as aldosterone, which affects arterial BP, but not HR. Moreover, cholesterol is the first building block of steroids, and citrate, the main substrate of mINDY, can be used to generate cholesterol. Next, we assessed whether *mIndy* deletion can reduce steroid hormone levels and synthesis. Circulating steroid hormone concentrations did not show significant differences between genotypes (Figure 7A), and microarray analysis showed similar expression levels of genes important in steroid hormone synthesis regardless of the genotypes. These results were also verified by RT-PCR (Figure 7B). Moreover, rate-limiting enzymes of sterol (StAR) and aldosterone synthesis (CYP11B2 or aldosterone synthase) were unaltered between genotypes (Supplemental Figure 2).

To address local vascular tone, vascular smooth muscle constriction in response to potassium and NE was measured ex vivo. Also, endothelial function was assessed by measuring maximum aortic ring relaxation using cumulative increasing concentrations of acetylcholine (ACh) in aortic rings precontracted by phenylephrine ex vivo (Supplemental Figure 4 and Supplemental Table 1). Aortic ring diameters and vessel tone were similar in the 2 genotypes (Supplemental Table 1). These data do not support the idea that changes in vascular structure or responsiveness contributed to changes in BP with *mIndy* deletion. Finally, developmentally induced structural changes in the heart itself can largely contribute to changes in BP and HR. We therefore assessed cardiac function in vivo by echocardiography in mINDY-KO, WT littermate, and additional weight-matched WT control mice. The last group was included because mINDY-KO mice showed a lower body length and weight compared with WT littermates, which may affect echocardiographic measurements (Supplemental Figure 1A and Supplemental Figure 5A). Of significance, in vivo echocardiography of mice under anesthesia led to similar HRs between the 3 experimental groups (Supplemental Figure 5B). In this setting, ejection fraction (Supplemental Figure 5C), stroke volume (Supplemental Figure 5D) and strain (Supplemental Figure 5, E and F), as well as fractional shortening, cardiac output, end-diastolic- and end-systolic volume, and end-diastolic and end-systolic left ventricle mass (Supplemental Figure 6, A–F) were similar between mINDY-KO and weight-matched WT control mice, used as the correct control group instead of WT littermates, indicating that there were no cardiac structural abnormalities associated with the deletion of *mIndy* and that no morphological changes accounted for the observed phenotype. Last, analysis of heart morphological structure by H&E staining was similar between mINDY-KO and WT littermate controls (Supplemental Figure 7).

## Discussion

In this study, deletion of the longevity gene *mIndy* (*Slc13a5*) lowered BP and HR as measured by radiotelemetry. Pharmacological and biochemical profiling revealed that *mIndy* affects long-term BP regulation through a potentially novel mechanism regulating sympathoadrenal tone and catecholamine biosynthetic pathways. Concomitant reductions in arterial BP and HR suggested that the response was mediated by a common mechanism. Indeed, the depressor response to ganglionic receptor blockade with trimethaphan was attenuated, indicating reduced sympathoadrenal signaling to the periphery. In addition, oscillations of systolic BP in the low-frequency band, which are mediated through the sympathetic nervous system (11), were reduced. Moreover, BRS was increased in mINDY-KO mice, which typically occurs with a shift of the balance between sympathetic and parasympathetic activity toward parasympathetic predominance in vivo (11, 23–25). The sympathetic nervous system regulates BP and HR via the release of catecholamines from adrenergic terminals, which can then be detected in 24-hour collected urine of mice. Catecholamine concentrations in the urine of mINDY-KO mice were reduced when compared with WT controls. Unbiased microarray analysis of mINDY-KO adrenal glands showed a reduction in the pathways involved in catecholamine synthesis, such as TH, the rate-limiting step in catecholamine biosynthesis. The results were confirmed by RT-PCR and immunoblotting. Citrate treatment increased global catecholamine biosynthesis in the adrenal pheochromocytoma cell line MPC, an effect that was partially blunted by inhibition of mINDY with a small molecule inhibitor, previously shown to reduce citrate uptake and to improve metabolic control in obese mice in vivo (21). Together, these data suggest that *mIndy* has an important function in cardiovascular control by reducing sympathoadrenal support of arterial BP.

Centrally generated efferent sympathetic activity is transmitted through postganglionic nerve fibers to peripheral organs, including blood vessels and the heart (26). Ganglionic blockade with trimethaphan virtually abolishes postganglionic adrenergic traffic (11, 27). The reduced depressor response to trimethaphan in mINDY-KO mice is consistent with attenuated tonic support of blood pressure through the sympathetic nervous system. Indeed, low-frequency band oscillations of arterial BP, which are mainly mediated through the sympathetic nervous system, were also reduced in mINDY-KO mice. Catecholamine-secreting cells including adrenal medullary cells convert tyrosine serially to l-DOPA and dopamine, which is converted further to NE and E; both are stored within secretory vesicles (28, 29).

Citrate can serve as a substrate for fatty acid synthesis, and the acetyl moiety of the intermediate acetyl-CoA can be used for histone acetylation, which is critical regulator of gene expression. Citrate is also known to inhibit glycolysis by inhibiting phosphofructokinase-1 and activate gluconeogenesis by stimulating fructose-1,6-bisphosphatase (30). Moreover, inhibition of citrate uptake by *mIndy* deletion results in AMPK activation and increased mitochondrial activity (8, 31). Mitochondrial respiratory activity is involved in several aspects of catecholamine synthesis and secretion in adrenal samples from patients with pheochromocytoma (32) but also in physiological adrenal responses to stress in mice (33). Overall, we hypothesize that the interaction between transcriptional epigenetic regulation and a change in cellular energy levels contributes to the effect of citrate on catecholamine synthesis and secretion.

In mice, cumulative systemic catecholamine secretion is best reflected by 24-hour collected urine measurements. Blood drawing in mice is associated with stress, leading to immediate release of stress hormones, such as catecholamines, that can confound the analysis of catecholamine secretion. In our study, 24-hour collected urine specimens showed that NE and E as well as their degradation products NMN and MN were robustly reduced in mINDY-KO mice, indicating a strong effect of *mIndy* on catecholamine synthesis and extraneuronal and adrenal medullary catecholamine metabolism. In line with these observations, we noticed relatively high *mIndy* expression in the adrenal medulla compared with other organs and tissues. Moreover, unbiased, whole-genome, microarray-based analysis of the adrenal glands of mINDY-KO and WT littermate controls showed reduced catecholamine biosynthetic processes. These findings were confirmed by RT-PCR and protein analysis of genes coding for several rate-limiting enzymes in catecholamine biosynthesis, TH, DDC, DBH, and PNMT. In contrast, expression of genes coding for adrenal catecholamine-degrading enzymes such as MAO and COMT were unaltered. Incubation of MPC cells with citrate, the main mINDY transporter substrate, markedly increased catecholamine level in these cells, an effect blunted by pharmacological inhibition of mINDY. Together, these data suggest that mINDY interacts with the sympathoadrenal system at least in part through catecholaminergic pathways.

Cardiovascular and metabolic diseases are tightly linked as exemplified by the metabolic syndrome, which comprises abdominal adiposity, altered glucose and lipid metabolism, increased BP, and often higher HR (34). Many individuals with obesity or type 2 diabetes are hypertensive, an observation consistent with a solid crosstalk between cardiovascular and metabolic control mechanisms. Deletion of *mIndy* has previously been shown to improve metabolic disease, such as diet-induced obesity, insulin resistance, and nonalcoholic fatty liver disease in mice (5, 8, 9, 22). The cardiovascular phenotype in our study implicates a role of *mIndy* in this cardiometabolic crosstalk. Reduction of BP in mINDY-KO mice could be mediated through direct mINDY actions or via indirect influences on BP control through changes in body composition. Here, mice were fed a normal chow diet, which excluded the contribution of body composition changes, specifically fat mass, in BP regulation between both genotypes. Confirming previous reports, mINDY-KO mice were slightly smaller and thus had reduced BWs (8). Otherwise, mINDY-KO mice showed similar proportions of fat and lean mass compared with their littermate controls as assessed with NMR in vivo. Therefore, differences in body composition do not explain our findings. Moreover, smaller animals tend to have higher HRs than larger ones, which is not seen in the mINDY-KO mice (35–38).

We also note that in mINDY-KO mice the vascular responsiveness toward adrenergic stimulation with phenylephrine was unaffected ex vivo, suggesting that the change in BP was mediated through altered catecholamine release rather than changes in vascular α-adrenoceptor responsiveness. Furthermore, potassium-induced vasoconstriction did not differ between mINDY-KO and WT littermate controls, indicating that smooth muscle constrictor functions were unchanged in KO mice. In vivo echocardiography revealed similar ejection fraction, stroke volume, and cardiac output and strain when HR was controlled by anesthesia and when mINDY-KO mice were compared with weight-matched WT control mice. These data indicate that deletion of *mIndy* is not associated with structural and/or functional abnormalities in the heart.

Our study cannot fully exclude that other mechanisms also contributed to changes in cardiovascular autonomic control. For example, reduced synthesis of steroid hormones such as aldosterone could contribute to the response. However, steroid hormone levels were not different between mINDY-KO and WT mice, and genetic as well protein levels of the major enzymes involved in steroid hormone synthesis were not changed. Because mINDY is expressed predominantly in the adrenal medulla, we hypothesize that catecholamines are more affected than steroid hormones. Reduced expression of catecholamine-synthesizing enzymes supports this idea. Our data on the pheochromocytoma cells show that citrate, the main mINDY substrate, increased catecholamine content, while pharmacological inhibition of citrate uptake blunted this effect, making developmental effects of mINDY on catecholamine synthesis unlikely.

We did not observe structural or functional changes in the heart with histological analysis of myocardial tissue or echocardiography in vivo. Of note, echocardiography was performed during anesthesia, which led to similar HRs between mice. An additional challenge, such as pressure overload or myocardial infarction, may be required to unmask influences of mINDY on cardiac structure. Future studies are needed to address this important point.

To exclude variations in physiological responses by hormonal fluctuations in female mice, we have used male mice throughout the study (39–44). The selection of male mice represents a limitation of our study, and more studies are needed to show that the observed effects are also present in female mice.

Reducing *Indy* gene expression leads to longevity in lower organisms and to a phenotype akin to caloric restriction in mice, an intervention that is well known to induce longevity in most species (6–8). Interestingly, reduced sympathetic activity and lower BP are associated with cardiovascular protection and prolonged life span (45, 46). Longevity studies in mINDY-KO mice will be extremely interesting. It will be important to determine whether the hypotensive effect of *mIndy* will be present also in aged mice and how this translates into cardiovascular morbidity and mortality.

Deletion or reduction of *mIndy*/mINDY has been shown to be protective against diet-induced metabolic disorders, including obesity, insulin resistance, and nonalcoholic fatty liver disease (5, 8–10). Obesity in general goes along with increased sympathetic activity (47–50). Here, we added potentially novel pieces to generate a more complete picture of the benefits of decreasing mINDY activity by showing that deletion of *mIndy* also affects arterial BP and HR by reducing sympathoadrenal activity. Remarkably, recent genome-wide association studies suggested that the Slc13a5 polymorphism rs16956192 contributes to variability in arterial BP (51). In addition to providing a potentially novel mechanism contributing to cardiometabolic crosstalk, our study further supports mINDY as a promising target for the whole spectrum of metabolic syndrome components, including increased BP.

## Methods

### Animals.

Mice were generated as described (8): pups carrying the *SLC13A5^tm1.1Helf^* allele were genotyped at the expected frequency of about 25%. Mice carrying *SLC13A5^tm1.2Helf^* allele (Δ1-6) were obtained by mating *SLC13A5^tm1.1Helf/+^* mice with animals carrying *Hprt1*-Cre transgene: 129S1-Hprttm1(Cre)Mnn (The Jackson Laboratory). The offspring was then backcrossed more than 10 times to the C57B/6J background, and studies were performed with age-matched littermate control WT mice. Homozygous mINDY-KO mice were obtained from mating heterozygous mINDY-KO mice. Littermate animals were used in all experiments; however, for echocardiography we also used WT mice (Charles River Laboratories) that were weight matched to mINDY-KO mice as a third experimental group. All breeding and procedures were carried out at the animal facilities of the Charité Berlin and Technical University Dresden according to institutional regulations and the local guide for the use and care of laboratory animals.

Experiments based on BP measurements by telemetry were performed on 12- to 16-week-old mINDY-KO male mice and littermate controls. We have selected male mice to exclude variations in physiological responses by hormonal fluctuations of estrogen and progesterone in female mice (39–44). The animals had free access to standard chow (0.25% sodium, SNIFF GmbH, Soest, Germany) and drinking water ad libitum. Animals were cared for within the Institutional Animal Care Committee guidelines, and all animal procedures were approved by local government authorities.

### Analysis of body composition.

Body composition was analyzed before and after BP studies using proton NMR (^1^H-MRS), and adipose tissue mass was quantified in vivo using micro-computed tomography. After studying telemetry, spectral analysis, baroreceptor HR reflex, sequence technique, BRS, pharmacological testing, and echocardiography (as described below), mice were anesthetized for the harvest of various tissues and organs including heart, aorta, adrenal glands, brain, skeletal muscle, epigonadal and subcutaneous adipose tissues, and liver.

### Telemetry.

Before implantation of the TA11PA-C20 BP device (Data Sciences International), the zero offset was measured, and the unit was soaked in 0.9% NaCl. Mice were anesthetized with isoflurane (Cura Med Pharma). The pressure-sensing catheter was advanced via the right femoral artery into the abdominal aorta, and the transmitter was placed in a subcutaneous pocket along the right flank. During surgery and in the recovery period, the mice were placed on a heated table to maintain body temperature. The mice were synchronized to a 12-hour light/12-hour dark schedule with lights on at 6:00 am. All mice were allowed a 7-day recovery from surgery before baseline BP and HR values were recorded for 3 days. By this time, the mice had regained their circadian BP and HR rhythms, and the surgery and anesthesia-dependent initial changes in BP and HR were followed by stabilization of both values. The mice received a normal chow diet and tap water (0.25% NaCl).

The data from the TA11PA-C20 device were transmitted via radio frequency signals to a receiver below the home cage and thereafter collected using the DATAQUEST ART system, version 2.1 (Data Sciences International), which allowed us to detect, collect, and analyze signals from several animals simultaneously. The data were sampled every 5 minutes for 10 seconds continuously with a sampling rate of 1000 Hz and stored on a hard disk. Systolic and diastolic BP, mean arterial pressure (MAP), and HR were recorded using the DATAQUEST software (ART 2.1). HR was computed from the pulse intervals of the BP recordings. For statistical analysis, we used 3 days of baseline values. Rhythm analysis for 27-hour periods in individual mice were performed using DQFIT program, which calculates the amplitude and the acrophase of both parameters (52). Activity was monitored as changes in transmitter signal strength due to the mouse (transmitter) locomotion. For further evaluation of cardiovascular function, the baroreceptor HR reflex was investigated using spontaneous changes in BP and HR. For this purpose, beginning after the 3 days of baseline values, BP waveforms were stored in a beat-by-beat modus 1 hour before and 1 hour after intraperitoneal injections of different drugs for pharmacological blockade of the autonomic nervous system. Beat-by-beat BP and HR values were obtained from these continuously recorded data.

### Autonomic blockade.

To evaluate autonomic control of BP in mINDY-KO and WT littermate control mice, continuous beat-by-beat values of BP and HR were recorded for 1 hour, after which the animals were administered the ganglionic blocker trimethaphan at a dose of 120 mg/kg intraperitoneally. Continuous beat-by-beat values of BP and HR were recorded for 1 additional hour. Handling caused an HR and MAP increase in both strains, which decreased over time following trimethaphan injection. Forty-five minutes after the injection of saline, MAP and HR were comparable to baseline values. Therefore, the values 1 hour after drug injection were used to characterize the trimethaphan response. Because of the drug’s pharmacodynamics, the effects were determined at the nadir of the HR and BP responses, between 7 and 12 minutes after injection (53). The time ranges for calculating HR and BP changes were the same. HR variability and baroreceptor function were also determined in steady-state conditions to exclude stress-induced BP and HR changes.

### Spectral analysis and BRS.

For evaluation of cardiovascular function, the baroreceptor HR reflex was investigated using spontaneous changes in BP and HR. The power spectra of systolic BP, pulse interval time series, and the cross spectra were calculated using fast Fourier transformation (FFT) (54–58). The power spectral density was estimated by the Welsh method with FFT length of 512 sample points, interpolation, resampling with 12 Hz, linear trend elimination, and Hanning window. The data analysis was performed with the PV-wave software (Visual Numerics). Five representative intervals were chosen and averaged according to the following criteria: *1*) steady-state conditions, *2*) no large and sudden BP changes, and *3*) no artifacts. The frequency bands were adapted for analysis in mice considering the ranges of HR and breathing frequencies (low frequency about 0.25–1.0 Hz, high frequency about 1.0–6.0 Hz). Low-frequency components of pulse interval spectrum (LF), high-frequency components of pulse interval spectrum (HF), LF/HF ratio, low-frequency power of systolic BP (SBP-LF), and root mean square of successive differences between adjacent normal pulse intervals were calculated. The baroreflex gain (BRS-LF) was determined as mean value of the transfer function between systolic BP and pulse intervals in the low-frequency band. BRS was considered significant if the coherence in the analyzed frequency band was about 0.8.

### MPC cell culture.

MPCs generated from heterozygous neurofibromatosis KO mice were acquired from Arthur Tischler (New England Medical Center and Tufts University School of Medicine, Boston, Massachusetts, USA) (59). MPC cells were seeded on collagen A–coated cell culture dishes (60) and maintained in RPMI-1640 medium containing 10% horse serum and 5% fetal calf serum supplemented with 2 mM GlutaMAX (complete medium). Collagen, RPMI, and GlutaMAX were from Thermo Fisher Scientific. Cells were cultured at 37°C, 5% CO_2_, and 95% humidity. MycoAlert Mycoplasma Detection Kit (Lonza, Switzerland) was used to ensure that cells were mycoplasma free. After trypsinization (trypsin/EDTA; 0.05%/0.02%) cells were diluted with complete medium and counted using the Neubauer counting chamber. All experiments were performed free of antibiotics for at least 1 passage after recultivation.

### Radiolabeled C^14^-citrate uptake in MPC cells.

^14^C citric acid (Hartmann, 114 μCi/mmol) was dissolved in uptake buffer (140 mmol/L NaCl, 1 mol/L KCl, 1 mol/L CaCl_2_, 1 mol/L MgSO_4_, 550 mmol/L glucose, 1 mol/L HEPES), and unlabeled citric acid was added to the final concentration mentioned. MPCs (50,000 cells/well, 48-well dishes) were incubated with the uptake solution in the absence or presence of 1 μM of the mINDY inhibitor PF-06761281 (Pfizer) for 10 minutes at 37°C as described (61). Cells were washed 3 times with ice-cold uptake buffer, lysed with 0.2% SDS. The intracellular radioactivity was detected by liquid scintillation counting (PerkinElmer Life Sciences). For each concentration 3 to 6 wells were used for each concentration of radiolabeled citrate. The specific mINDY-mediated citric acid uptake was obtained by subtracting off the nonspecific uptake measured in the presence of PF-06761281.

### Catecholamine measurements in MPCs.

MPCs were seeded in 24-well plates at a density of 1.5 × 10^5^ cells/well (4 replicates for each condition). Once attached, cells were treated with vehicle (control), 5 mM sodium citrate, or 5 mM sodium citrate combined with 1 μM of the INDY inhibitor PF-06761281 (Pfizer). Cells were incubated for 72 hours, then washed with PBS, and 3 wells per condition were extracted by adding 100 μL 0.4 M perchloric acid containing 0.5 mM EDTA. The remaining well was used for normalization by counting the corresponding cell number (Neubauer chamber). After centrifugation to remove cell debris, supernatants were stored at –80°C. Catecholamines were analyzed by liquid chromatography with electrochemical detection as described previously (62).

### MPC proliferation, cell viability, and apoptosis assays.

For evaluation of cell proliferation, cells were counted by using the Neubauer chamber 24, 48, and 72 hours after seeding. CellTiter AQueous One Solution Cell Proliferation Assay (Promega) was used to determine the number of viable cells. For analyzing programmed cell death, we employed the Caspase 3/7 Assay (Promega), which provides a profluorescent substrate with an optimized bifunctional cell lysis/activity buffer for caspase-3/7 (DEVDase) activity assays (60). All assays were performed according to the manufacturer’s protocols and guidelines.

### Ex vivo analysis of vascular function.

Vascular function was analyzed in aortic segments using a Mulvany myograph (56). First, the basal tone in the 2 experimental groups (mINDY-KO and WT littermate controls) was measured after applying 20 mmHg distension to aortic segments. Vessels were preconstricted with a potassium-enriched solution or phenylephrine (PE). Endothelial function was analyzed in aortic rings after precontraction with 0.3 mmol/L PE and subsequent relaxation in response to increasing concentrations of ACh. The level of ACh-induced relaxation mediated by nitric oxide (NO) was determined using NO synthase inhibitor l-NAME. To analyze vascular smooth muscle relaxation, the NO donor sodium nitroprusside was added to PE-precontracted aortic segments.

Vascular function was assessed as described previously (63, 64). In brief, the thoracic aortas were excised and cleaned of adherent fat and connective tissue. The length of proximal aortic segments was determined using a calibrated ocular micrometer. Aortas were mounted on 2 pins (200 mm diameter) in a Mulvany myograph 410A (DMT A/S). One pin was attached to an isometric force transducer; the other was attached to a micrometer screw, allowing for control of the inner diameter of the vessel preparation. All segments were continuously gassed with carbogen (5% CO_2_, 95% O_2_) in Krebs-Henseleit buffer in the presence of 10 mmoL/L diclofenac. Temperature was set to 37°C. Data are presented as sigmoidal concentration response curves (GraphPad Prism 6.0, GraphPad Software, Inc.). Effects of ACh are expressed as percentage PE-induced precontraction.

### Echocardiography.

Transthoracic echocardiography in all experimental groups (WT *n* = 5, mINDY-KO *n* = 6, weight matched *n* = 5) was performed in anesthetized animals (2% isoflurane) via an oxygen mask. Respiration, temperature, and echocardiography were continuously monitored during the whole scans. Rectal temperature was maintained at approximately 38°C by a heated platform. Hair was removed from the abdomen by a clipping machine and depilatory cream. Prewarmed gel was used as an ultrasound-coupling medium. Cardiac function and morphology were assessed by echocardiography with a Visual Sonics Vevo 770 High-Resolution Imaging System with the use of a high-resolution (40 MHz) transducer. Stroke volume and cardiac output were measured by tracing the endocardium in systole and diastole of a parasternal long-axis view of the left ventricle. All images were acquired and stored for off-line analysis by a blinded observer using Visual Sonics Vevo Strain software (Version 2.2.0). B-Mode cine loops were used in parasternal long view to assess basic parameters for systolic function (ejection fraction, fractional shortening, end-diastolic volume, end-systolic volume, stroke volume, cardiac output, and left ventricular mass) and more accurate and robust speckle tracking analysis (global longitudinal strain, global longitudinal strain rate). Negative values illustrate fiber shortening of the myocardium. Images were checked for quality with regard to differentiation of wall borders and absence of artifacts. Left ventricle endocardium was traced manually in parasternal long-axis views in end-diastole. The epicardium was automatically traced by the software, checked, and manually adjusted if necessary for maximum tracking accuracy. Analysis was performed on 3 consecutive cardiac cycles, and mean values from the 3 measurements were calculated. Global strain values were obtained from the average of the 6 segments of the left ventricle.

### Catecholamine profiling by liquid chromatography‑tandem mass spectrometry.

To determine urinary catecholamine, metanephrine, and steroid levels, a subset of WT (*n* = 9) and mINDY-KO (*n* = 9) mice were housed in individual metabolic cages. After 1 day of adaptation, urine was collected for 24 hours over the next 2 days. Only animals with comparable urine volume were included in the study for further analysis.

Measurements of steroid hormones were batch analyzed by liquid chromatography‑tandem mass spectrometry (LC‑MS/MS) as described previously (65). Urinary catecholamines as NE and E and their O-methylated free metabolites NMN and MN were simultaneously determined by LC-MS/MS, as described previously (66). For the latter, urinary osmolality was used for correction of different sample volumes. Quantification of hormone levels was done by comparing ratios of analyte peak area obtained from samples to the respective peak area of stable isotope-labeled internal standard calibrators.

### Adrenal gland and heart histology.

Adrenal glands and hearts of mINDY-KO (*n* = 3) and WT littermate control mice (*n* = 3) were fixed in 4% buffered paraformaldehyde and embedded in paraffin. Then, 3 × 5 μm sections per tissue were placed on 1 slide and stained with H&E. Whole adrenal gland and heart were analyzed using the mosaic function of ApoTome microscope Observer.Z1 (Carl Zeiss). Images were taken at 10×, 20×, and 40× original magnification.

### Gene expression.

Microarray (NCBI Gene Expression Omnibus datavase accession number GSE124886) and RT-PCR techniques were carried out according to standard procedures to determine the effect of mINDY-KO on gene expression in mouse adrenal glands. Full methodological details are described in Supplemental Methods.

### Statistics.

Data are presented as means ± SEM. If not stated otherwise, statistically significant differences in mean values were evaluated by Student’s *t* test (2 tailed) and ANOVA (2 way). Slopes and intercepts of data sets were tested for significance using Microsoft Excel and GraphPad Prism. The method is equivalent to an analysis of covariance (ANCOVA). *P* values of less than 0.05 were considered statistically significant. Outliers were detected using the 2-sided Grubb’s method.

### Study approval.

All animal experiments were approved by the local government authorities (Landesamt für Gesundheit und Soziales, Berlin, Germany, and Landesdirektion Sachsen, Dresden, Germany).

## Author contributions

DMW, SL, JJ, and ALB designed research studies. DMW, SL, AK, ACDCG, MP, AH, WK, KK, MW, NB, MB, JT, and ALB performed experiments and analyzed data; SLH provided mINDY-KO mice; AK, TS, CH, AK, NNEA, DNM, HM, AD, AEA, RC, GE, JJ, and MD analyzed data; DMW, JJ, and ALB wrote the manuscript; and SRB, AD, SLH, RC, and GE edited the manuscript. DMW, MD, AK, SL, NB, TS, CH, NNEA, ACDCG, MP, AH, WK, KK, DNM, HM, AD, MW, AEA, SLH, SRB, RDC, MB, GE, JT, JJ, ALB read and approved the final version of the submitted manuscript.

## Supplementary Material

Supplemental data

## Figures and Tables

**Table 1:**
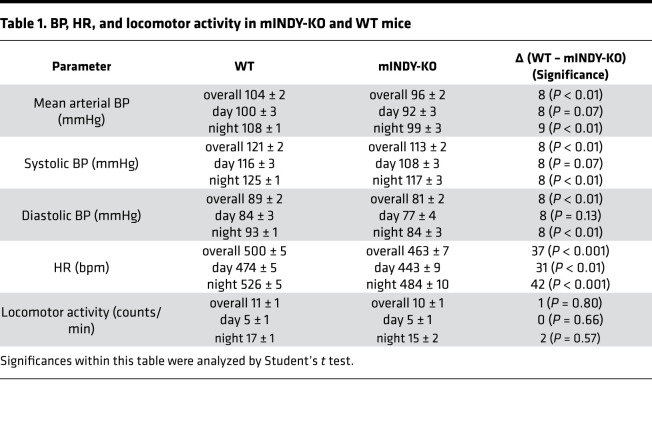
BP, HR, and locomotor activity in mINDY-KO and WT mice

## Version 1 01/25/2021

Electronic publication
