# Supplementary material for: The longevity gene mIndy (I’m Not Dead, Yet) affects blood pressure through sympathoadrenal mechanisms
Source: JCI Insight. 2021 Jan 25;6(2):e136083. doi: 10.1172/jci.insight.136083 (PMC7934862; doi:10.1172/jci.insight.136083)
Supplement: Supplemental data [file jciinsight-6-136083-s098.pdf]

Supplemental data

Willmes et al., The longevity gene *mIndy* affects blood pressure through sympathoadrenal mechanisms.

Supplemental figures

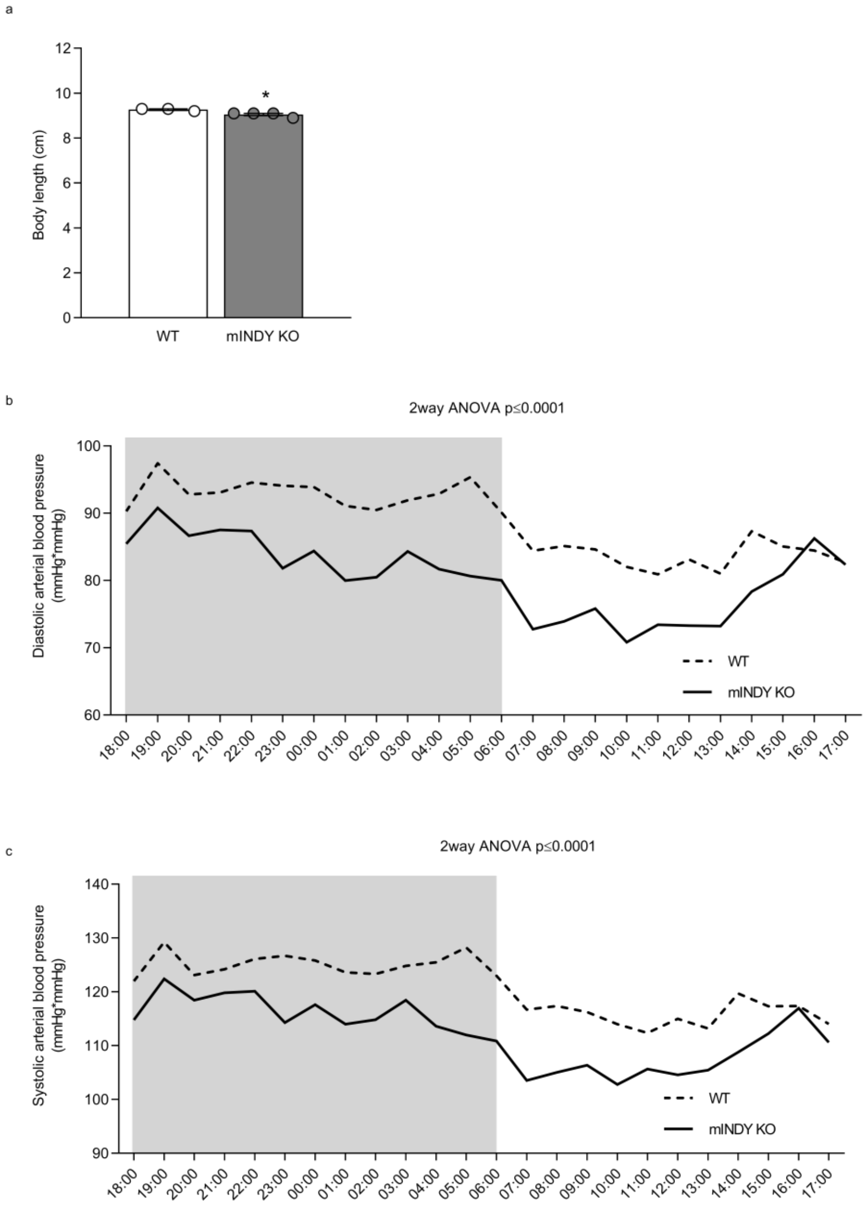

Supplementary figure 1. Body length and arterial BP of mINDY KO mice and WT littermates.

(**a**) Body length of mINDY KO mice and WT littermate controls (WT n = 3; 9.3±0.03cm; mINDY KO n = 4; 9.1±0.05cm) at the age of 13-14 weeks fed a regular chow diet. Results were determined by a two-sided student´s ttest. Data represent the mean ±SEM (*P<0.05). (**b**) Diastolic and (**c**) systolic arterial blood pressure of mINDY KO and WT littermate control mice. Results were determined by a two-way ANOVA (***P<0.001).

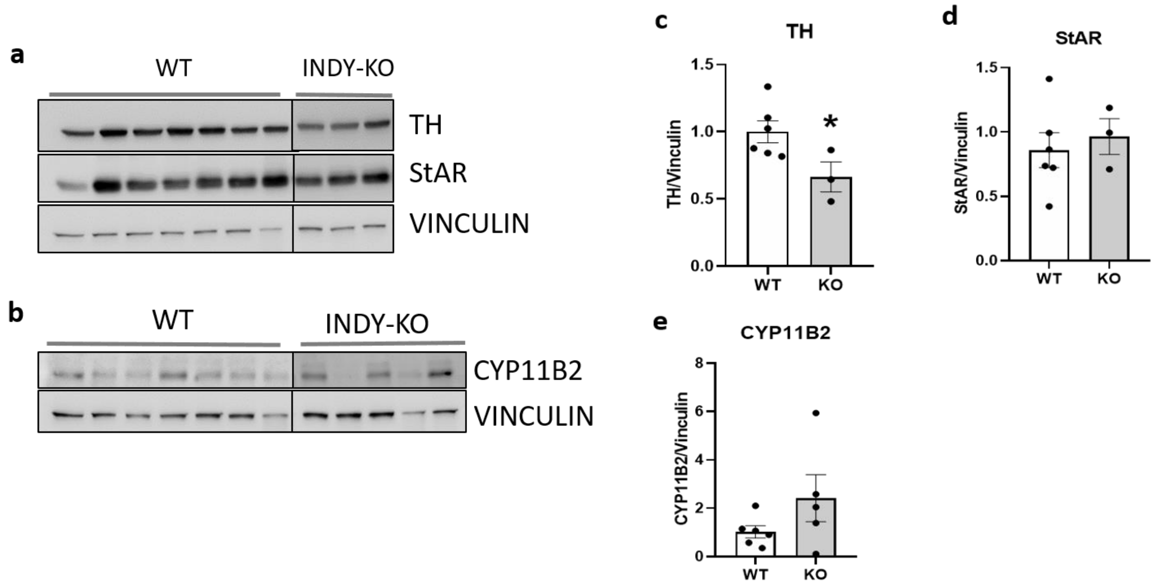

Supplementary figure 2. Western Blot analysis of TH, StAR and CYP11B2 in adrenal glands of mINDY KO mice. Protein expression by western blot analysis and quantification of TH, StAR (a, c, d) and CYP11B2 (b, e) in adrenal glands of mINDY KO (n=3) and WT control mice (n=7). Proteins were normalized to the protein expression of vinculin.

TH=tyrosine hydroxylase, StAR=steroidogenic acute regulatory protein, CY11B2=cytochrome P450 family 11 subfamily B member 2.

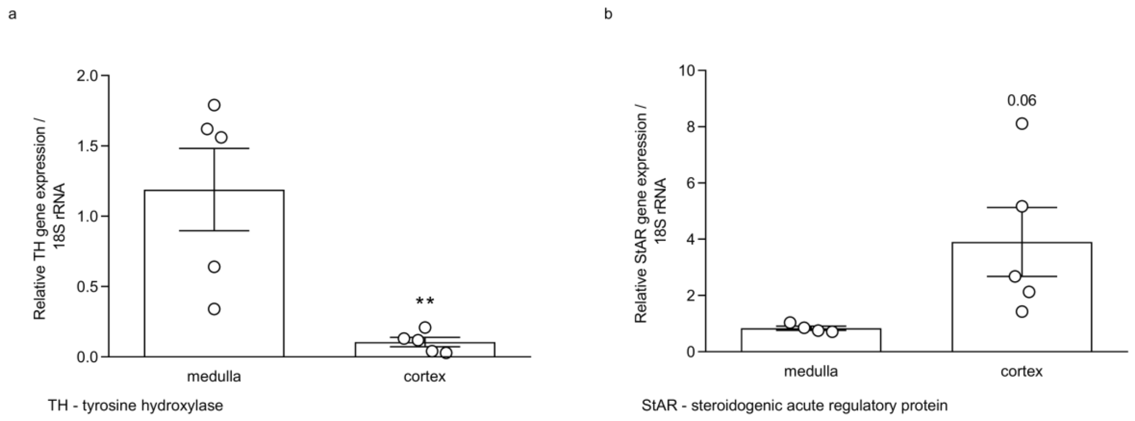

Supplementary figure 3. Confirmation of effective separation of adrenal medulla from cortex. (**a**) Tyrosin hydroxylase (TH) gene expression in adrenal medulla (*n* = 5) and cortex (*n* = 5) of WT mice fed a regular chow diet. (**b**) Steroidogenic acute regulatory protein (StAR) gene expression in adrenal medulla (*n* = 4) and cortex (*n* = 5) of WT mice fed a regular chow diet. Results were determined by a two-sided student´s ttest. Data represent the mean ±SEM (***P*<0.01;****P*<0.01).

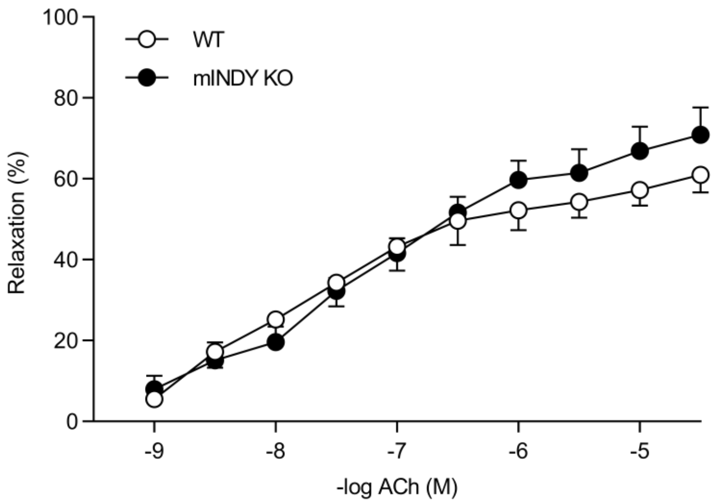

Supplementary figure 4. Endothelial function in aortae of WT and mINDY KO mice.
Endothelial function in aortae of WT (*n* = 8) and mINDY KO (*n* = 8) mice. Endothelial function was assessed using cumulative increasing concentrations of acetylcholine (ACh) in phenylephrine-precontracted aortic rings. Data represent the mean ±SEM.

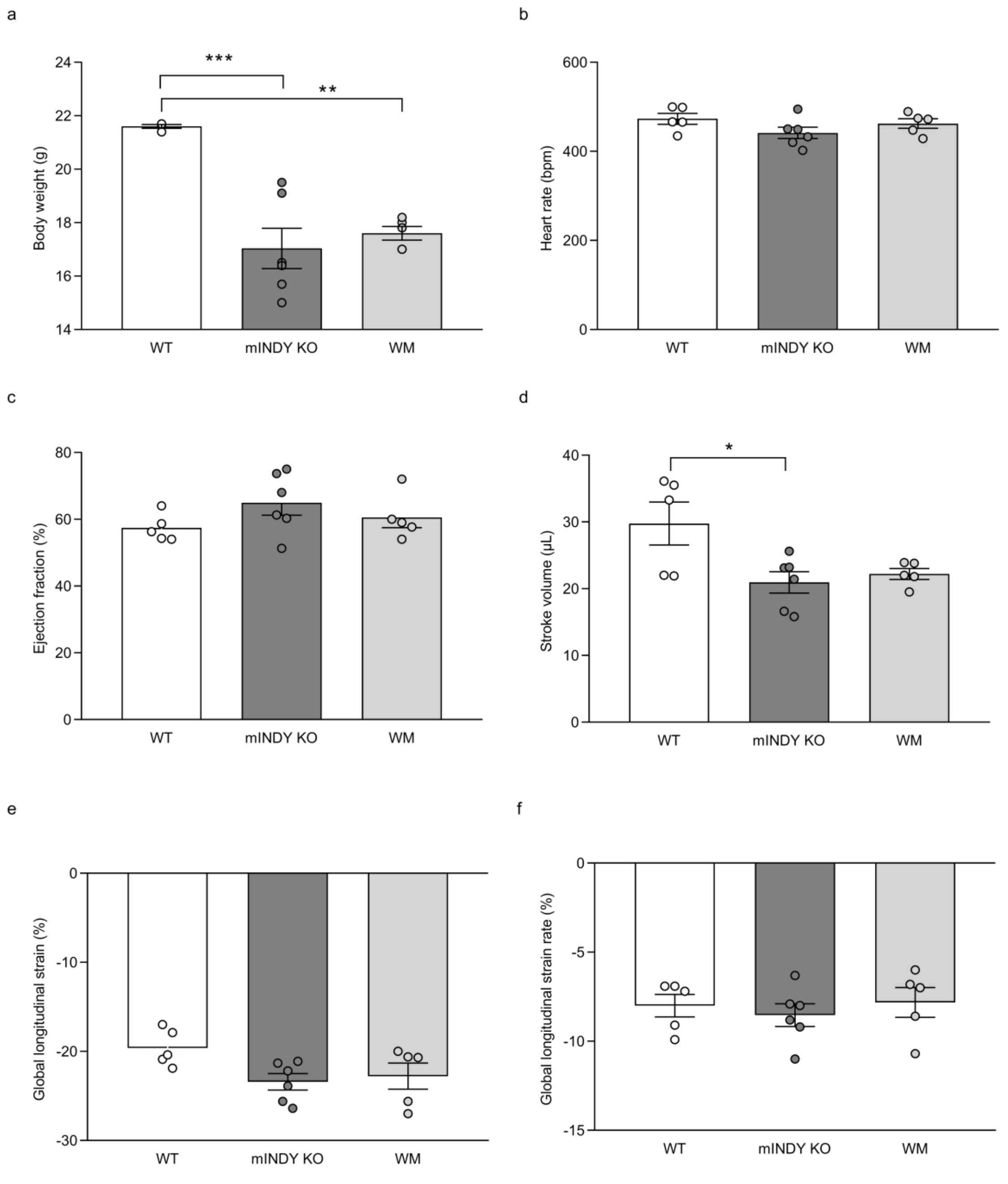

Supplementary figure 5. Cardiac parameters in mINDY KO mice, WT littermate controls, and weight-matched (WM) control mice.

Cardiac parameters in mINDY KO (*n* = 6), WT littermate controls (*n* = 5), and weight-matched WT mice (*n* = 5) were measured by echocardiography and MRI. (**a**) Body weight was measured in all three groups prior to experiment. (**b**) Heart rate was kept similar in all three groups of mice under anesthesia. (**c**) Ejection fraction is presented as % and (**d**) stroke volume as volume of blood pumped from the left ventricle per heartbeat. (**e**) Global longitudinal strain and (**f**) global longitudinal strain rate are presented as %. Significance level was determined by one-way ANOVA. Data represent the mean ±SEM (*$P<0.05$; **$P<0.01$; ***$P<0.001$).

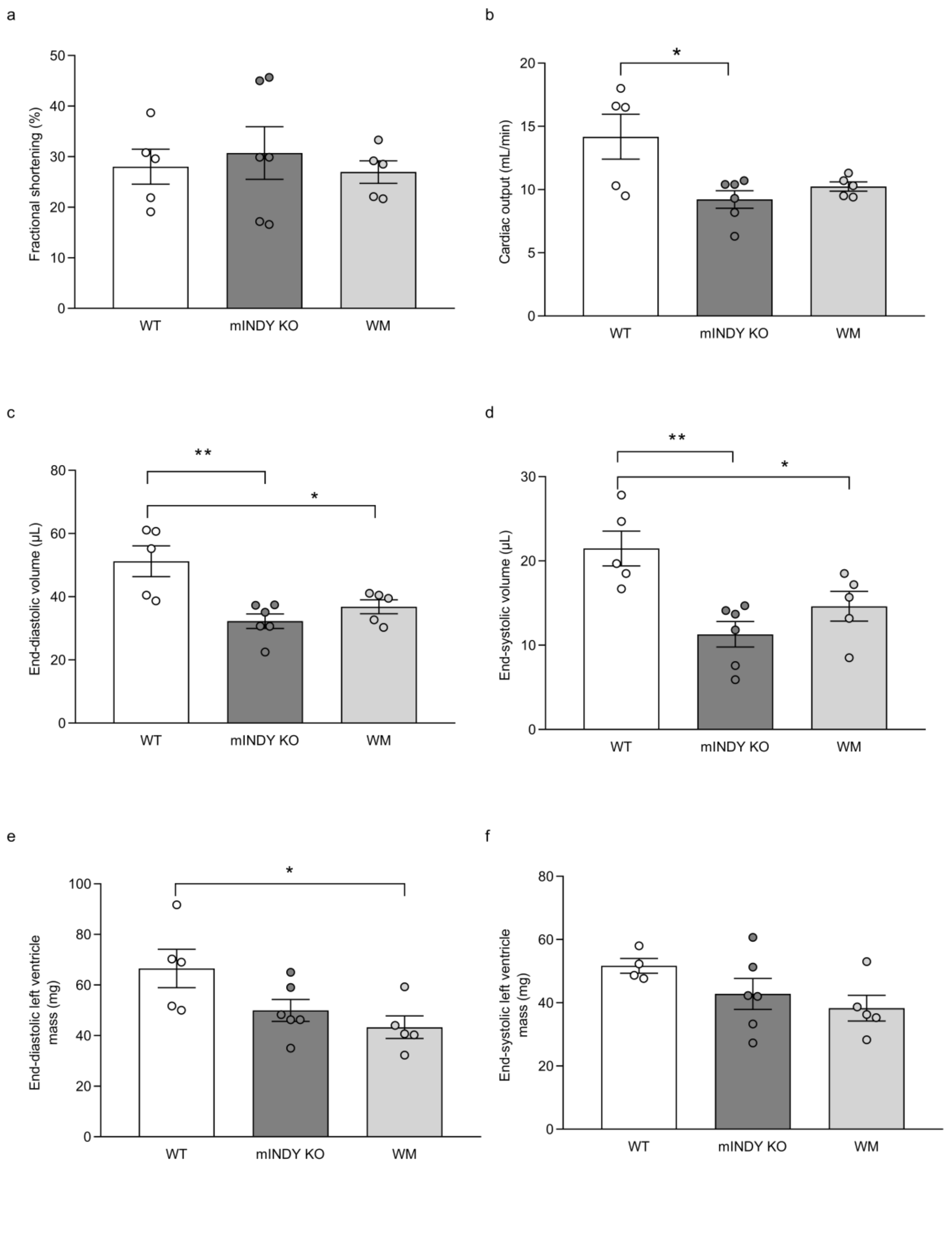

Supplementary figure 6. Additional cardiac parameters of mINDY KO mice and WT littermate controls and weight-matched (WM) control mice.
Cardiac parameters of mINDY KO (n = 6) and WT littermate controls (n = 5) and weight-matched WT mice (n = 5) were measured by echocardiography and MRI. (**a**) Fractional shortening, (**b**) cardiac output, (**c**) end-diastolic and (**d**) end-systolic volume, (**e**) end-diastolic and (**f**) end-systolic left ventricle mass. Results were determined by a one-way ANOVA. Data represent the mean ±SEM (*P<0.05; **P<0.01).

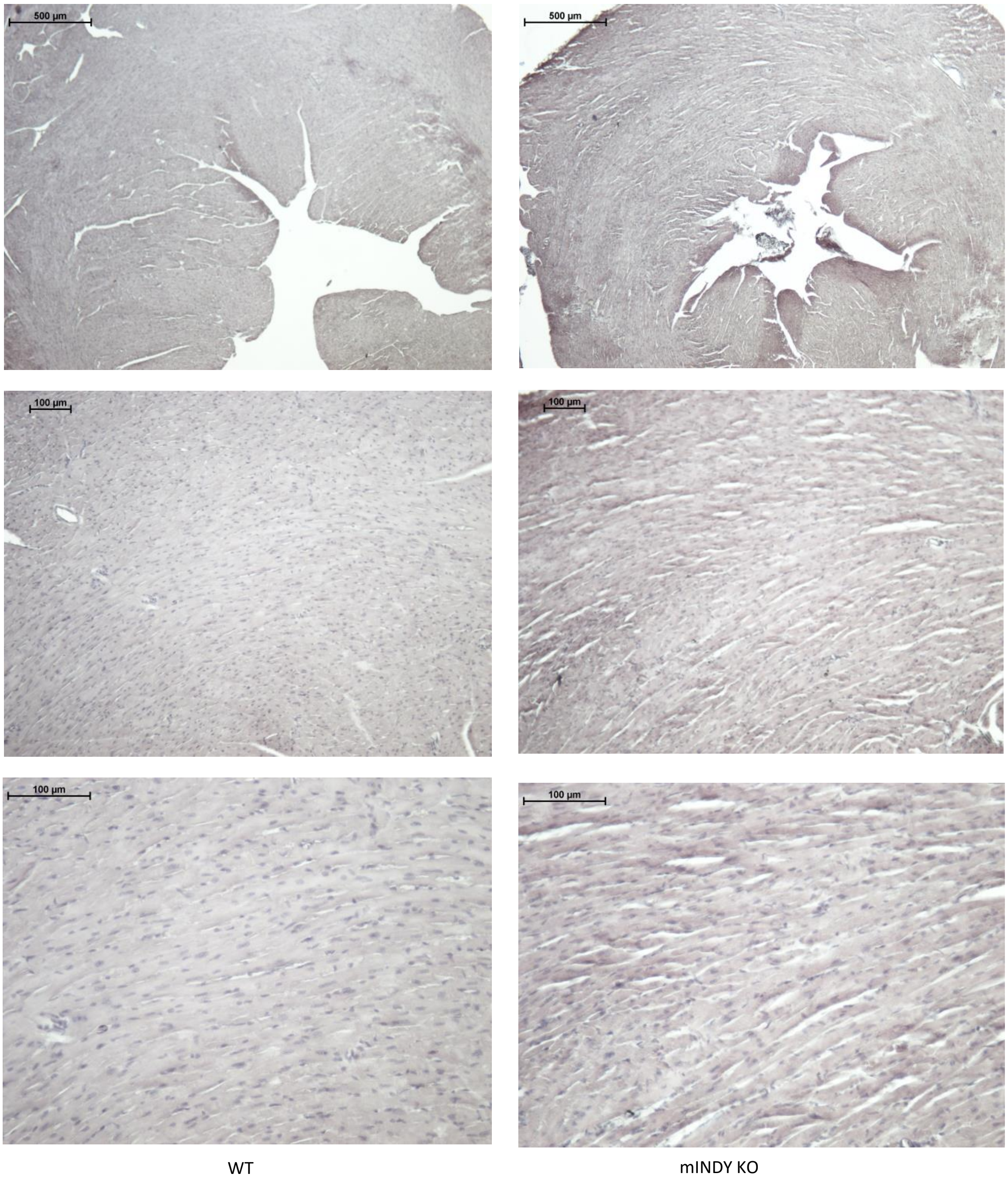

Supplementary figure 7. Heart morphological structure of WT and INDY KO mice.
Analysis of heart morphological structure by H&E staining of mINDY KO and WT littermate controls fed a regular chow diet. Shown are representative microscopy staining pictures of both genotypes, three magnifications (10x, 20x, 40x) each.

Supplemental tables

Supplementary Table 1. Characterization of aortic diameter and function.
Summarized characterization of aortic diameter, vessel tone and relaxation in aortae of mINDY KO (*n* = 7) and WT littermate control mice (*n* = 7). Data represent the mean ±SEM. (KPSS: potassium enriched physiological saline solution (123 mM K^+), L-NMMA: L-N- monomethyl-arginine; unpaired T-test).

Parameter	mINDY KO	WT	Significance
Diameter (µm)	618 ± 25	667 ± 22	n.s.
Vessel tone (mN/mm)			
- KPSS	0.91 ± 0.06	0.98 ± 0.08	n.s.
- Phenylephrine	0.84 ± 0.09	0.82 ± 0.10	n.s.
- Phenylephrine + L-NMMA	1.69 ± 0.16	1.59 ± 0.14	n.s.
Relaxation (%)			
- Acetylcholine	71 ± 7	61 ± 5	n.s.
- Acelylcholine in presence of L-NMMA	3 ± 5	4 ± 4	n.s.
- Sodium-nitroprusside	106 ± 3	102 ± 2	n.s.

Supplemental Methods

Microarray analysis

RNA was extracted from mouse adrenals (mINDY KO, *n* = 5; WT, *n* = 6; normal chow diet) using the Trizol reagent (Invitrogen, Carlsbad CA) according to standard protocols. RNA quality and quantity were determined using the Agilent RNA6000 Bioanalyzer chip. Five-hundred ng total RNA was labeled and hybridized to Illumina BD-202-0202 expression bead chips using standard Illumina protocols. Following standard post-hybridization rinses, blocking and staining, the fluorescent hybridization signal was quantified on an Illumina iScan scanner and analyzed using Illumina Genome Studio Software (v 2011.1, Gene expression module 1.9.0). Raw microarray data were subjected to Z-normalization; individual genes with Z ratio > 1.5 in both directions, ANOVA *P* value < 0.05, and false discovery rate < 0.30 were considered significantly changed as described (1, 2). Gene Ontology analysis and parametric analysis of gene set enrichment (PAGE) method were conducted using tools that are part of DIANE 6.0 software (http://www.grc.nia.nih.gov/branches/rrb/dna/diane_software.pdf) (3). For each Z (pathway), a *p* value was computed in JMP 6.0 to test for the significance of the Z score obtained.

RNA extraction, cDNA synthesis, and quantitative PCR

Total RNA was extracted from mouse adrenal glands and purified using Trizol reagent (Invitrogen) following the manufacturer's instructions (mINDY KO, *n* = 12-13; WT littermate controls, *n* = 15). First-strand cDNA was synthesized from 1 ug RNA using High-Capacity reverse transcription kit with random hexamers, according to the manufacturer's instructions (Applied Biosystems, Foster City, CA). "iTaq™ Universal SYBR® Green Supermix" (Bio-Rad, Hercules, CA) was used for quantitative PCR reactions, and samples were analyzed on the Quant Studio 6 Flex (Applied Biosystems). Oligonucleotide sequences were as follows: TH, 5'CAAGGTTCCCTGGTTCCCAA-3' and 5'-TTGGCTCACCCTGCTTGTAT-3'; DDC, 5'GGCTTACATCCGAAAGCACG-3' and 5'-

TCACTGATGTGTTCCCAGGC-3’; DBH, 5’TGCACCACATGGAGGTCTTC-3’ and 5’-CCTGCCGGCCTTGTATCTTC-3’; PNMT, 5’GTAGCTGTCGCCTTGGCTTA-3’ and 5’-GACACCTCACCGGTAGCAAA-3’; MAO, 5’GCGGTACAAGGGTCTGTTCC-3’ and 5’-CAGCCAATCCTGAGATGCCG-3’; COMT, 5’TGGTGGCTATTGGTTGGTTTG-3’ and 5’-CCATTCGCACGGCTGAGTA-3’. The PCR reactions were performed by melting temperature analysis. The relative gene expression was calculated by the ΔΔCT method and normalized using the housekeeping gene HPRT. 5’ACTTCGAGAGGTCCTTTTCACC-3’ and 5’-GCAAACTTTGCTTTCCCTGG-3’. *mIndy* gene expression was measured using the quanti tect primer assay (Qiagen).

Adrenal cortex and medulla isolation

Adrenal glands were first explanted from mice, fried from surrounding fat and kept in a sterile phosphate-buffered saline (PBS) on ice. Adrenals were then put on Petri dishes in sterile ice-cold PBS and the medulla was carefully cut out of the surrounding cortex with a safety scalpel (Brown) under the dissection microscope as described previously (4-6). Only clearly visual separated medulla and cortex were collected in pre-chilled separate Eppendorf tubes containing RLT- Plus RNA extraction buffer (RNeasy Micro Kit) for further RNA isolation.

Adrenal cortex and medulla RNA extraction, cDNA synthesis, and quantitative PCR

RNA isolation was performed using RNeasy Micro Kit and reverse transcribed using iScript RT Kit (Biorad). As a marker of good separation between cortex and medulla, the expression of Star and TH genes were evaluated by Q-PCR (Syber Green; Qiagen and Roche 2.1. PCR Machine) (5). Oligonucleotide sequences were as follows: TH, 5’AAGGGCCTCTATGCTACCCA-3’ and 5’GCCAGTCCGTTCCTTCAAGA-3’; StaR, 5`TTGGGCATACTAACAACCA-3’ and 5´GAAACACCTTGCCCACATCT-3; 18S rRNA, 5’GTTCCGACCATAAACGATGCC-3’ and 5´TGGTGGTGCCCTTCCGTCAAT-3’.

Western Blot analysis

Tissue was lysed in RIPA buffer (50 mM Tris-HCl, pH 7.4, 150 mM NaCl, 1 % 599 Nonidet P-40, 0.5 % sodium deoxycholate, 0.1 % SDS) containing protease and phosphatase inhibitors (Life Technologies). 10-50 µg protein lysate was separated on 10 % SDS-PAGE gels and transferred to methanol-activated PVDF membranes using the wet electrophoretic transfer system (Bio-Rad). Membrane was blocked with 5 % BSA or milk powder in TBS/0.1 % Tween, primary antibodies were incubated at 4 °C overnight and secondary antibodies for one hour at room temperature. Peroxidase-conjugated secondary antibodies and Western Bright Chemiluminescence Substrate Sirius (Biozym) were used for protein visualization using the Fusion FX (Vilber Lourmat) and Fusion FX7 Advance imaging software. Densitometric analysis was carried out using ImageJ software (NIH). The following antibodies have been used: anti-Vincullin (Abcam, ab129002, 1:10000), anti-GAPDH (Abcam, ab8245, 1:20000), anti-StAR (Cell Signaling, 8449, 1:1000), anti-TH (Sigma Aldrich, AB152, 1:2000), anti-CYP11B2 (kindly provided by the lab of Prof. Celso E. Gomez-Sanchez, Medical Center of the University of Mississippi, USA), goat anti-rabbit IgG, HRP 615 26 conjugated (Millipore, 401353, 1:10000), goat anti-mouse IgG, HRP conjugated (Millipore, 616 401253, 1:10000). Mice were between 13 and 26 weeks of age.

Supplemental references

1. Cheadle C, Vawter MP, Freed WJ, and Becker KG. Analysis of microarray data using Z score transformation. *J Mol Diagn.* 2003;5(2):73-81.
2. Lee JS, Ward WO, Ren H, Vallanat B, Darlington GJ, Han ES, et al. Meta-analysis of gene expression in the mouse liver reveals biomarkers associated with inflammation increased early during aging. *Mech Ageing Dev.* 2012;133(7):467-78.
3. Kim SY, and Volsky DJ. PAGE: parametric analysis of gene set enrichment. *BMC Bioinformatics.* 2005;6:144.
4. Rubin de Celis MF, Garcia-Martin R, Wittig D, Valencia GD, Enikolopov G, Funk RH, et al. Multipotent glia-like stem cells mediate stress adaptation. *Stem Cells.* 2015;33(6):2037-51.
5. Fliedner SM, Breza J, Kvetnansky R, Powers JF, Tischler AS, Wesley R, et al. Tyrosine hydroxylase, chromogranin A, and steroidogenic acute regulator as markers for successful separation of human adrenal medulla. *Cell Tissue Res.* 2010;340(3):607-12.
6. Santana MM, Chung KF, Vukicevic V, Rosmaninho-Salgado J, Kanczkowski W, Cortez V, et al. Isolation, characterization, and differentiation of progenitor cells from human adult adrenal medulla. *Stem Cells Transl Med.* 2012;1(11):783-91.
